# Use of upadacitinib for recalcitrant oral erosive lichen planus

**DOI:** 10.1016/j.jdcr.2025.02.042

**Published:** 2025-03-28

**Authors:** Connor A. Sheehan, Nicholas D. Brownstone, Jason B. Lee, Sylvia Hsu

**Affiliations:** a Department of Dermatology, Temple University Hospital, Philadelphia, Pennsylvania; b Department of Dermatology and Cutaneous Biology, Thomas Jefferson University, Philadelphia, Pennsylvania

**Keywords:** erosive oral lichen planus, upadacitinib

## Introduction

Oral lichen planus (LP) is a T-lymphocyte-mediated autoimmune destruction of oral keratinocytes and basement membrane disruption with no clear causative antigen.^1^ The erosive form of oral LP presents with individual or coalescing painful ulcerations in the oral mucosa.^2^ Topical corticosteroids, such as triamcinolone acetonide or clobetasol propionate, are recognized as the first-line treatment of oral erosive LP, but these topical agents have limited efficacy and are mainly used for symptomatic relief.^2^ There are no known effective systemic therapies for oral erosive LP, and this condition is both difficult to control and debilitating for patients. In this case report, we describe a successful use of upadacitinib in the treatment of oral erosive LP.

## Case Report

**Fig 1:**
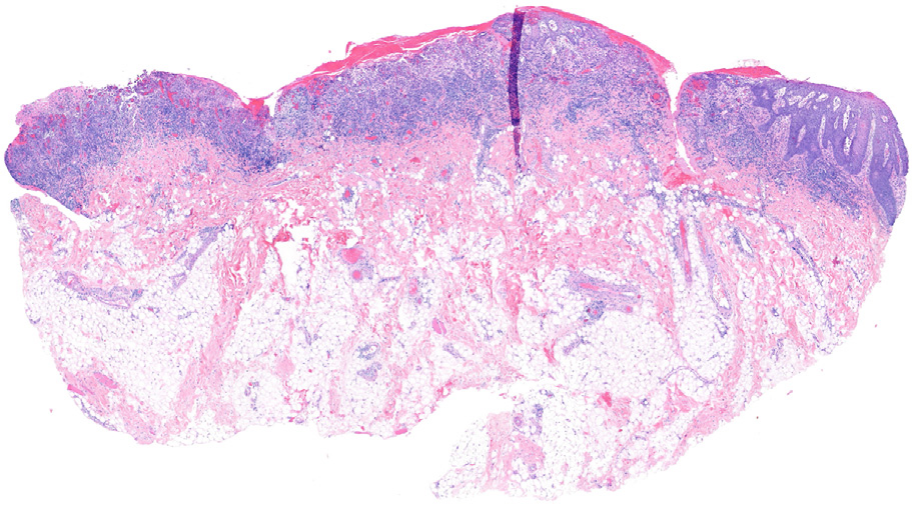
The incisional biopsy of the right buccal mucosa shows a dense band-like lymphoplasmacytic infiltrate within the upper submucosa with broad zones of erosion and focal ulceration (H&E 40×).

**Fig 2:**
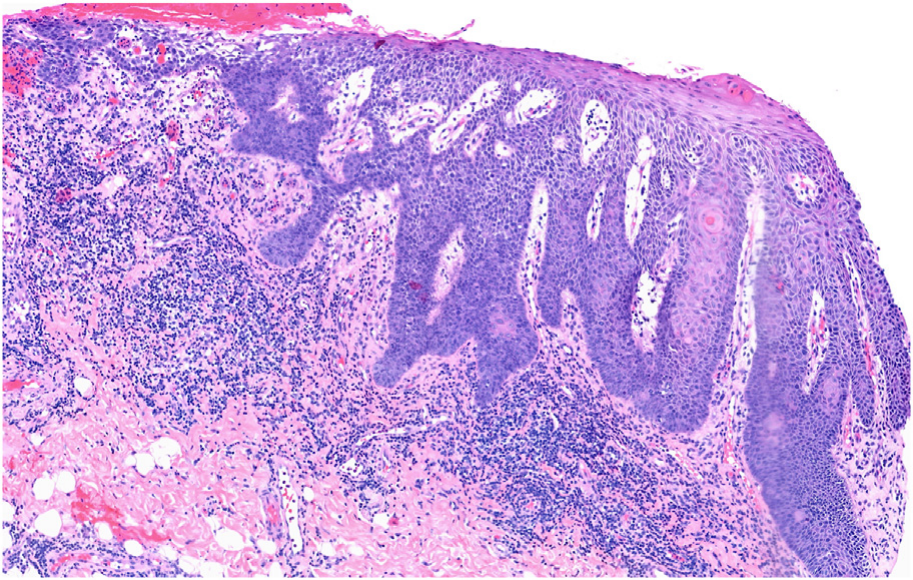
The dense lichenoid infiltrate is accompanied by irregular hyperplasia of the epithelium with rare scattered epithelial cells near the basal layer (H&E 100×).

**Fig 3:**
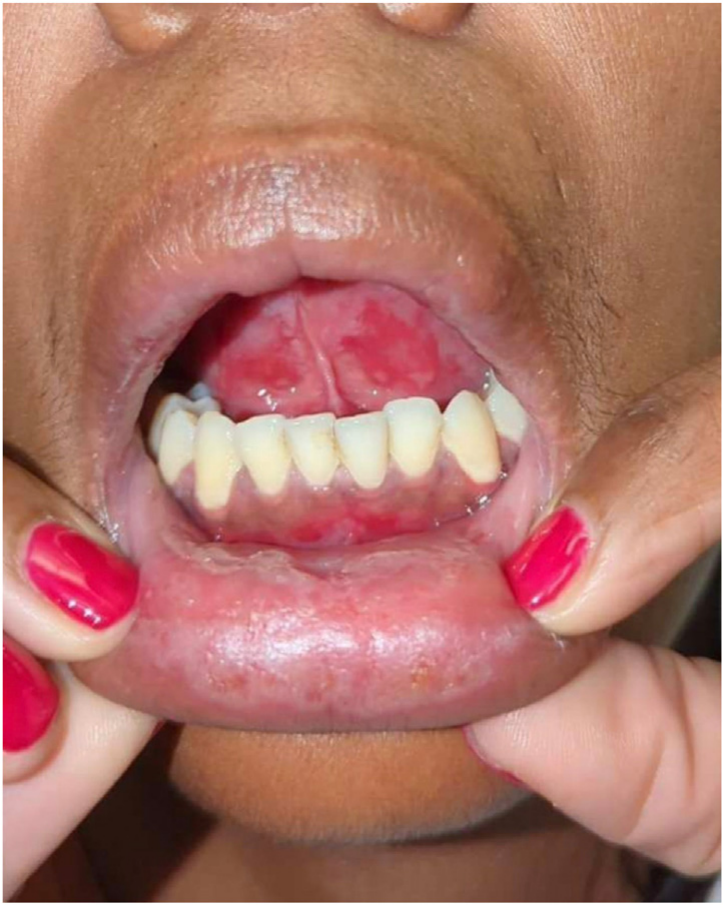
Erosions on the gingiva and the underside of the tongue.

A 39-year-old African American woman with a past medical history of recurrent herpes simplex virus infection presented to the dermatology clinic after an exacerbation of long-standing painful oral ulcers. The symptoms began 4 years before her presentation to us with 2 biopsies taken from the right buccal mucosa by oral surgery: one for histopathology and one for direct immunofluorescence. The histopathologic findings showed a dense band-like lymphoplasmacytic infiltrate within the upper submucosa with zones of erosion and focal ulceration. The intact areas of the epithelium showed irregular hyperplasia with rare scattered necrotic epithelial cells near the basal layer along with reactive changes due to the ulcer (Figs 1 and 2). The overall histopathologic findings are consistent with long-standing erosive LP. Direct immunofluorescence was negative. Prior to presentation, she had been unsuccessfully treated with a steroid mouthwash, oral prednisone, and chlorhexidine gluconate mouthwash, leading to recurrent flares, significant pain, avoidance of oral intake, and weight loss. On examination, oral erosions surrounded by a lacy white border were present on the buccal mucosa, gingiva, mucosal lips, and on the underside of the tongue (Fig 3). Laboratory studies were negative for hepatitis C and HIV and the enzyme-linked immunosorbent assay testing for desmoglein 1 and 3 antibodies was also negative.

**Fig 4:**
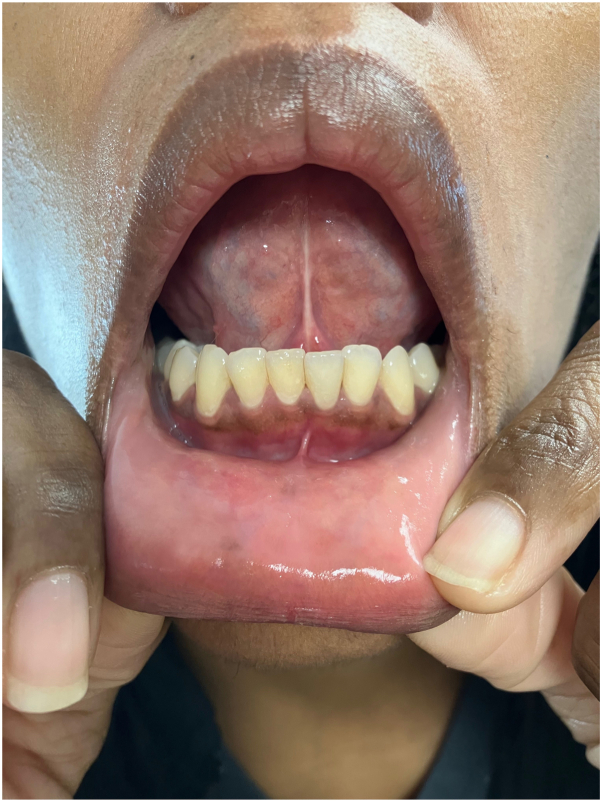
No evidence of erosive oral lichen planus after 4 weeks of upadacitinib 15 mg orally or by mouth daily.

Therapeutic management began with a 2-week course of oral prednisone 60 mg every morning with mild improvement, although pain persisted and the condition recurred. The patient was then started on mycophenolate mofetil 1000 mg twice a day, leading to a reduction in the frequency of flares for 13 months, although gastrointestinal side effects were significant and ulcers persisted. Mycophenolate mofetil was eventually discontinued, and the patient was switched to apremilast 30 mg twice a day for a total of 16 months. Again, she experienced decreased flare frequency but presented again with a severe flare consisting of multiple persistent erosions in the mouth. With recurrent flares of painful ulcers and inability to tolerate adverse reactions that emerged, the patient was prescribed upadacitinib 15 mg daily. At a 4-week follow-up visit, the patient showed complete clearance of oral ulcerations (Fig 4). The patient reported she had experienced minor headaches and worsening of her existing herpes simplex virus infection while taking upadacitinib. She described her headaches as mild and was prescribed daily prophylactic valacyclovir 500 mg daily for herpes simplex virus prophylaxis. At the 12-week follow-up, the patient remained clear while receiving upadacitinib 15 mg daily.

## Discussion

Oral erosive LP can be a debilitating disease for patients and has proven to be difficult to adequately treat because of a limited understanding of the exact pathogenesis. Our patient trialed several different topical and systemic medications over several years with inadequate efficacy and frequent disease flares. Although the mechanism of oral erosive LP has not yet been fully elucidated, Janus kinase (JAK) inhibitors, such as upadacitinib, may be efficacious in broadly targeting signaling pathways responsible for inflammatory skin conditions. Data have shown that CD8^+^ tissue-resident memory cells contribute specifically to the erosive form of oral LP, with a significant increase in the release of interferon gamma compared with immune cells found in nonerosive oral LP.^3^ Upadacitinib predominantly inhibits the JAK1 enzyme,^4^ which is phosphorylated in part by cytokine receptors sensitive to interferon gamma, and thus offers a specific target for therapy in oral erosive LP. Although upadacitinib is currently US Food and Drug Administration approved for use in rheumatoid arthritis, psoriatic arthritis, atopic dermatitis, ankylosing spondylitis, Crohn disease, ulcerative colitis, and nonradiographic axial spondylarthritis,^4^ a small number of case reports have described varying levels of treatment for oral LP symptoms in patients who were resistant to conventional treatment options.^5^, ^6^, ^7^, ^8^ Kooybaran et al^6^ were the first to report the use of upadacitinib as monotherapy for oral erosive LP, finding complete resolution after 24 weeks of treatment. Balestri et al^5^ found that upadacitinib led to a complete resolution of 3-week-old oral erosive LP lesions in a patient with concurrent psoriatic arthritis within just 7 days. Most recently, Slater et al^8^ described a patient, who was refractory to many treatments, experiencing 70% resolution of oral LP lesions after 4 weeks of upadacitinib therapy. All cases described in the literature used the same dose of upadacitinib, although some were confounded by concurrent use of additional anti-inflammatory medications, such as methotrexate.^7^ Table I summarizes the reported uses of upadacitinib for oral LP to date.

**Table I:** Reported uses of upadacitinib in oral lichen planus

Authors	Dose	Time to resolution
Balestri et al^5^	15 mg once daily	7 d
Kooybaran et al^6^	15 mg once daily	24 wk
Landells et al^7^	15 mg once daily	Not specifically mentioned. Complicated by oral squamous cell carcinoma.
Slater et al^8^	15 mg once daily	70% resolution after 4 wk

JAK inhibitors have been described to be at least partially effective in reducing symptom burden in a few cases of oral LP, although the data are limited.^5^, ^6^, ^7^, ^8^, ^9^ The case described in this article not only represents the third reported complete resolution of oral erosive LP lesions due to upadacitinib treatment but also indicates the potential side effects experienced with its use. There are no meta-analyses or randomized control trials in the literature evaluating the use of JAK inhibitors in oral LP. However, the individual reports may represent the emergence of a potential effective therapy. Further research is needed to determine the extent of efficacy for JAK inhibitors, such as upadacitinib, in the treatment of oral erosive LP as well as the side effect profile among this specific subset of patients.

## Conflicts of interest

None.
